# Involvement of an ABI-like protein and a Ca^2+^-ATPase in drought tolerance as revealed by transcript profiling of a sweetpotato somatic hybrid and its parents *Ipomoea batatas* (L.) Lam. and *I*. *triloba* L.

**DOI:** 10.1371/journal.pone.0193193

**Published:** 2018-02-21

**Authors:** Yufeng Yang, Yannan Wang, Licong Jia, Guohong Yang, Xinzhi Xu, Hong Zhai, Shaozhen He, Junxia Li, Xiaodong Dai, Na Qin, Cancan Zhu, Qingchang Liu

**Affiliations:** 1 Food Crop Institute, Henan Academy of Agricultural Sciences, Zhengzhou, China; 2 Key Laboratory of Sweetpotato Biology and Biotechnology, Ministry of Agriculture, China Agricultural University, Beijing, China; Purdue University, UNITED STATES

## Abstract

Previously, we obtained the sweetpotato somatic hybrid KT1 from a cross between sweetpotato (*Ipomoea batatas* (L.) Lam.) cv. Kokei No. 14 and its drought-tolerant wild relative *I*. *triloba* L. KT1 not only inherited the thick storage root characteristic of Kokei No. 14 but also the drought-tolerance trait of *I*. *triloba* L. The aim of this study was to explore the molecular mechanism of the drought tolerance of KT1. Four-week-old *in vitro*-grown plants of KT1, Kokei No. 14, and *I*. *triloba* L. were subjected to a simulated drought stress treatment (30% PEG6000) for 0, 6, 12 and 24 h. Total RNA was extracted from samples at each time point, and then used for transcriptome sequencing. The gene transcript profiles of KT1 and its parents were compared to identify differentially expressed genes, and drought-related modules were screened by a weighted gene co-expression network analysis. The functions of ABI-like protein and Ca^2+^-ATPase, two proteins screened from the cyan and light yellow modules, were analyzed in terms of their potential roles in drought tolerance in KT1 and its parents. These analyses of the drought responses of KT1 and its somatic donors at the transcriptional level provide new annotations for the molecular mechanism of drought tolerance in the somatic hybrid KT1 and its parents.

## Introduction

Sweetpotato (*Ipomoea batatas* (L.) Lam.) is an important food, fodder, industrial raw material, and bio-energy resource crop, and plays important roles in food security and energy security worldwide, especially in China [1]. In the context of the increasing world population and climate change, the global water shortage has become one of the major challenges in agricultural production. Agricultural activities account for about 75% of global water consumption; therefore, the impact of drought stress on the productivity of field crops is an important issue. Drought has become a major threat to sustainable crop cultivation [2, 3], as it significantly reduces the productivity of crops, including sweetpotato, causing huge economic losses [4, 5].

Selecting new, drought-tolerant sweetpotato varieties is one of the main strategies to cope with drought. However, there are several difficulties in the genetic improvement of sweetpotato, including the narrow genetic background, heterosis recession, and the lack of excellent gene resources [6]. Compared with cultivated varieties of sweetpotato, their wild relatives have accumulated and retained many unique and excellent genetic resources (such as drought tolerance) under harsh survival, competition, and natural selection conditions. Therefore, the wild relatives of sweetpotato could serve as a source of desirable genes to improve cultivated sweetpotato varieties and broaden their gene pool [7, 8]. However, the long history of reproductive isolation limits the ability of sweetpotato cultivars to cross with their wild relatives. This severely restricts the use of beneficial wild resources to generate new varieties by sexual hybridization. Somatic hybridization between sweetpotato and its wild relatives has opened a new breeding pathway to overcome interspecific and intraspecific cross-incompatibility, and provides a method to utilize the excellent genes in the wild species [1, 9].

Previously, we obtained the somatic hybrid KT1 by somatic hybridization between the sweetpotato cultivar Kokei No.14 and its drought-tolerant wild relative *I*. *triloba* L. Genomic *in situ* hybridization (GISH) confirmed that the chromosomes of KT1 were derived from Kokei No.14 and *I*. *triloba* L. [10]. KT1 not only inherited the thick storage root characteristic of Kokei No.14 but also the drought-tolerance trait of *I*. *triloba* L. [9, 10]. Next-generation sequencing technology has developed rapidly in recent years, and is now a fast, efficient, and high through-put sequencing method. The continuous development and improvement of this technology has provided new ideas and methods for research on functional genomics and whole transcriptomes [11–13]. Transcriptome sequencing has been used to explore the molecular mechanisms of various phenotypes, including the drought tolerance of sweetpotato and its wild relatives [14–17].

In this study, the differential expression profiles of the somatic hybrid KT1 and its parents were constructed by *de novo* transcriptome sequencing, and key genes related to drought tolerance were further enriched by a weighted gene co-expression network analysis (WGCNA), a lately developed approach to find modules of highly correlated genes which are allowed to be associated with phenotypic traits [18, 19]. The internal molecular mechanism of drought tolerance was systematically explored. The results of this study not only lay the foundation for clarifying the molecular mechanism of drought tolerance in KT1 and its parents, but also provide new ideas and gene resources for further theoretical research and breeding programs.

## Materials and methods

### Plant materials

Three different materials were used: Kokei No. 14 (K14), KT1, and *I*. *triloba* L. (Tri). K14 is a hexaploid sweetpotato cultivar (2n = 6x = 90) mainly grown in Japan and China. Tri is a diploid wild relative (2n = 2x = 30) with remarkable drought tolerance, but no storage root. KT1 is the somatic hybrid of K14 and Tri, which has a storage root like K14 and drought tolerance like Tri.

### Drought treatment

KT1 and its two parents were cultured on Murashige and Skoog (MS) solid medium under the following conditions: 27 + 1°C, 13-h light/11-h dark photoperiod, with a light intensity of 54 μmol m^-2^ s^-1^. After being subcultured for 4 weeks, the plants were transferred into ½-strength Hoagland’s solution containing 30% PEG6000 and treated for 6 h, 12 h and 24 h under the conditions described above. Untreated samples (0 h) served as the control. Samples were collected at each time point, quickly frozen in liquid nitrogen, and then stored at -80°C until use. Two biological replicates were established.

### RNA extraction and Illumina sequencing

Total RNA was extracted from whole plants using a Quick RNA Isolation Kit (Huayueyang Biotech Co., Ltd., Beijing, China) according to the manufacturer’s instructions. Residual DNA was removed by RNase-free Dnase I (TaKaRa Biotech Co., Ltd., Dalian, China), and RNA integrity was checked by electrophoresis on a 1.2% agarose gel. The RNA concentration was quantified by an Agilent 2100 Bioanalyzer (Agilent Technologies, Inc., Santa Clara, CA, USA). High-quality RNA samples were sent to the Biomarker Technologies Corporation (Beijing, China) for cDNA library construction and sequencing, which was performed using the Illumina HiSeq 2500 platform with 125-bp paired-end reads.

### Data filtering and *de novo* assembly

High-quality clean reads were obtained after quality control by removing adaptor sequences, duplicated sequences, ambiguous reads (‘N’), and low-quality reads (that had N bases of more than 10% and that bears more than 50% of bases that have a Q-value<10). Trinity (version: r20131110; http://trinityrnaseq.sourceforge.net/) was used for transcriptome *de novo* assembly after data normalization of the clean reads. Clean reads with a certain overlap length were initially combined to form long fragments without N (contigs). Related contigs were clustered using TGICL (2.1) software [20] to yield unigenes (without N) that could not be extended at either end. Redundancies were removed to acquire non-redundant unigenes. It should be noted that the unigene mentioned in this article is based on Trinity assembly, not equvalent to that based on EST sequences.

### Unigene functional annotation and expression calculation

The unigene sequences were compared with NR (Sep-21-2011), Swiss-Prot (Jan-07-2015), GO (Dec-12-2014), COG (Feb-08-2009), KOG (Feb-08-2009), and KEGG (Sep-21-2011), using BLAST (2.2.31) software [21], and KEGG orthologies were obtained using KOBAS (2.0) [22]. Unigene annotation information was obtained using HMMER (v3.0) software [23], the Pfam (27.0) database [24] and Araport11 database (Release_201606) after prediction of the unigene amino acid sequences.

For each sample, the reads were compared with those in the unigene library using Bowtie (1.1.1) [25], and transcript abundance was estimated according to the results of the comparison with RSEM (r2013-02-25) [26]. The counts were further transformed to FPKM (fragments per kilobase per million sequenced reads) values to compare transcript abundance among samples [27].

### Screening of differentially expressed genes

The transcript levels of unigenes in KT1 and its parents under drought stress for 6, 12 and 24 h were compared with those in the control (0 h). Gene expression data were filtered by removing genes with low transcript levels in all 24 samples. The standard for screening was that the FPKM value in at least one group of two biological replicates was ≥ 5. A total of 22,412 unigenes remained after screening. The remaining genes were used to identify the differentially expressed genes (DEGs), which were then analyzed by a weighted gene co-expression network analysis (WGCNA). The EBSeq (1.6.0) package was used to obtain the “base mean” value to identify DEGs. The absolute value of log_2_ (FPKM+1) ratio ≥ 1 and *p* value < 0.05 were set as the thresholds for a significant difference in gene expression between two biological repeats.

### Weighted gene co-expression network analysis

The FPKM values of the 22,412 unigenes filtered from the above FPKM screening were added by 0.001 and then normalized by a Log_10_ transformation (Lg (FPKM+0.001)). The WGCNA R software package (v1.41.1) was used to identify modules containing genes that were co-expressed and correlated with drought tolerance. Unsigned, weighted correlation network construction and module detection were performed using the automatic one-step function (for blockwise modules). The resulting Pearson correlation matrix was transformed into a matrix of connection strengths using a power of 10.

The resulting gene modules were assigned colors by R software and Module-Trait relationships were calculated by Pearson’s correlation analyses. Each module was represented by module Eigengenes, which were calculated from the first principal component capturing the maximum amount of variation of the module. Then, the topological overlap was calculated to measure network interconnectedness.

### Homologous cloning of candidate genes and evolutionary analysis

Two candidate genes screened from the WGCNA modules were further analyzed by Sanger sequencing. Primers were designed using Primer Premier 6 (http://www.premierbiosoft.com/) software, and homologous genes were cloned from K14, KT1 and Tri. Protein sequences of the cloned genes were compared with their homologs from other plant species to build a phylogenetic tree with MEGA 6.0 software using the neighbor-joining method [28].

### Accession numbers

The accession numbers for phylogenetic analysis in this article from the GenBank/EMBL databases are as follows: *Physcomitrella patens* (XP_001783354.1), *Selaginella moellendorffii* (XP_002981240.1), *Ananas comosus* (XP_020098808.1), *Brachypodium distachyon* (KQK08797.1), *Oryza sativa* (XP_015650826.1), *Setaria italica* (XP_004961070.1), *Zea mays* (XP_008656080.1), *Solanum lycopersicum* (NP_001333966.1), *Solanum tuberosum* (XP_006339648.1), *Vitis vinifera* (XP_002279419.1), *Manihot esculenta* (OAY62223.1), *Populus trichocarpa* (XP_002301384.2), *Gossypium raimondii* (XP_012475123.1), *Cucumis sativus* (XP_004133838.1), *Glycine max* (XP_003548613.1), *Malus domestica* (XP_008375925.1), AtABIL1 (NP_566067.1), AtABIL2 (NP_190498.1), AtABIL3 (NP_001318635.1), AtABIL4 (NP_001330354.1), AtABI1 (NP_194338.1), AtABI2 (NP_200515.1), Ca^2+^-ATPase 1 (NP_849716.1), Ca^2+^-ATPase 2 (NP_195479.1), Ca^2+^-ATPase 3 (ABU53680.1), Ca^2+^-ATPase 4 (Q9XES1.2),_Ca^2+^-ATPase 7 (O64806.2), Ca^2+^-ATPase 8 (Q9LF79.1), Ca^2+^-ATPase 9 (Q9LU41.2), Ca^2+^-ATPase 10 (Q9SZR1.2), Ca^2+^-ATPase 11 (Q9M2L4.1), Ca^2+^-ATPase 12 (Q9LY77.1), and Ca^2+^-ATPase 13 (Q9LIK7.1).

## Results

### Summary of transcriptome sequencing data

After quality control, 677,222,430 clean reads were obtained. The average clean reads per sample were 28,217,601.25, and the lowest was 24,146,171. The average Q30 base percentage was 90.91% and the lowest was 88.22% (S1 Table). A “total gene number vs total read number plot” was made to assess the quality of assembly and the depth of sequencing, which showed that sequencing volume was already saturated (S3 Appendix). All clean data were uploaded in the NCBI Sequence Read Archive database under the BioProject PRJNA413661 with SRA accession number SRR6169925-SRR6169948. After *de novo* assembly, 322,803 transcripts and 105,959 unigenes were gained. The sequence lengths were mainly distributed in the size range of 200–2000 bp (Table 1). The transcript and unigene N50 lengths were 1,965 bp and 1,403 bp, respectively, with 148,263 (83,496 + 64,767) transcripts and 23,083 (13,328 + 9,755) unigenes longer than 1 kb, respectively (Table 1).

**Table 1:** Statistics of splicing results of transcriptome sequencing data.

Length range	Contig	Transcript	Unigene
200–300 (bp)	4,246,309(97.87%)	52,286(16.20%)	36,300(34.26%)
300–500 (bp)	39,049(0.90%)	51,427(15.93%)	26,075(24.61%)
500–1000 (bp)	29,683(0.68%)	70,827(21.94%)	20,501(19.35%)
1000–2000 (bp)	15,206(0.35%)	83,496(25.87%)	13,328(12.58%)
2000+ (bp)	8,427(0.19%)	64,767(20.06%)	9,755(9.21%)
Total number	4,338,675	322,803	105,959
Total length (bp)	298,071,025	402,763,792	82,261,193
N50 length (bp)	64	1,965	1,403
Average length (bp)	68.70	1247.71	776.35

### Functional annotation of unigenes

The BLAST parameter E-value was set to < 1e-5 and the HMMER parameter E-value was set to < 1e-10, resulting in 36,767 unigenes with annotated information. The statistical results of gene annotations are listed in S2 Table.

### Gene expression analysis

**Fig 1:**
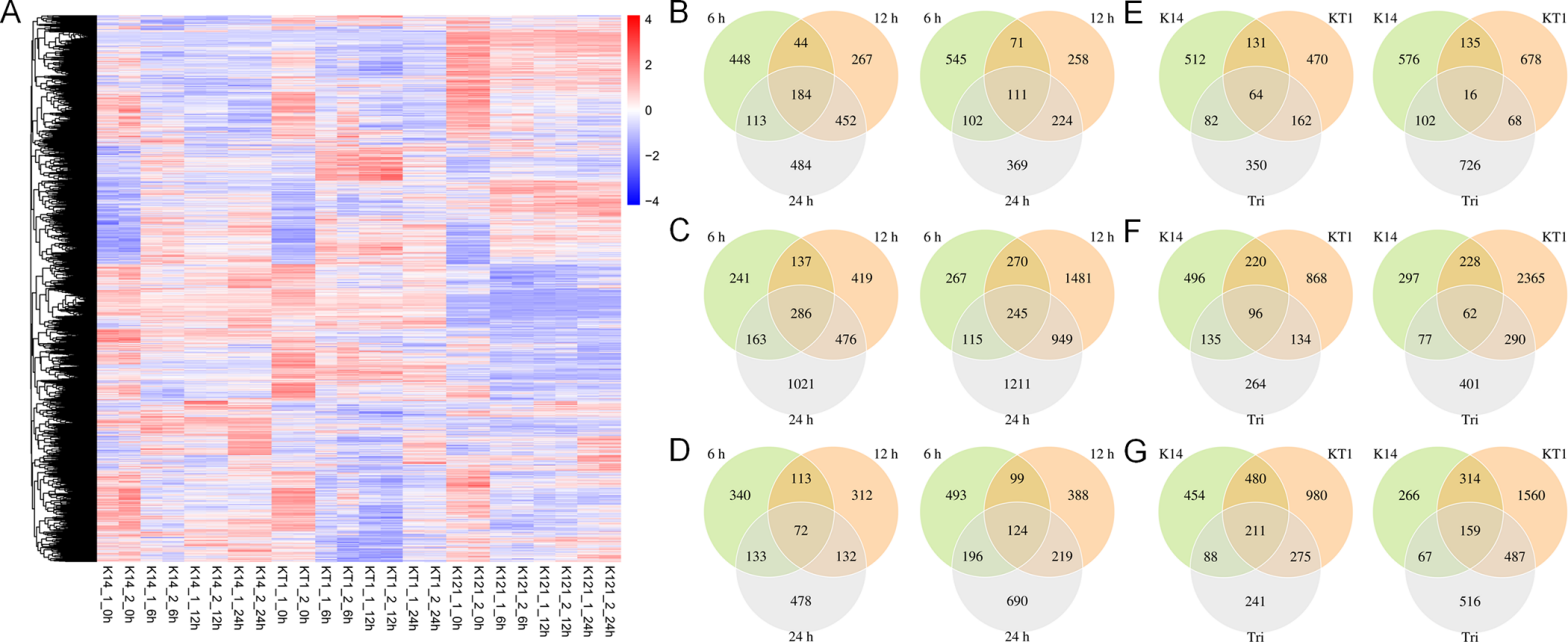
Heatmap clustering of unigenes and Venn diagram of differentially expressed genes (DEGs) in 24 samples. (A) Heatmap clustering of unigenes in 24 samples. (B-D) Venn diagrams of up-regulated and down-regulated DEGs under drought stress at 6 h, 12 h and 24 h in K14 (B), KT1 (C) and Tri (D). (E-G) Venn diagrams of up-regulated and down-regulated DEGs under drought stress in K14, KT1 and Tri at 6 h (E), 12 h (F) and 24 h (G). Left, up-regulated DEGs; right, down-regulated DEGs.

The gene expression data were screened to remove unigenes with low transcript levels in all 24 samples (12 groups). Finally, a total of 22, 412 unigenes were retained and used to construct the heatmap. The heatmap clustering indicated that the gene expression profiles were similar in the two biological repeats in each group, confirming high consistency between the two biological replicates (Fig 1A). At 0 h, the gene expression profiles of K14, KT1 and Tri were similar. After drought stress (6 h, 12 h, 24 h), the expression profiles differed among the three lines, with the greatest differences between K14 and Tri (Fig 1A). In KT1, the expression profiles of some unigene clusters were similar to those in K14, and the expression profiles of other unigene clusters were similar to those in Tri (Fig 1A).

The KT1 had the largest number of DEGs (1,724+4,263+4466), followed by K14 (1,618+1,611+2,039) and the wild relative Tri (1,570+1,459+2,044) (Table 2). At each time point (6 h, 12 h, and 24 h) during drought stress, there were more down-regulated DEGs than up-regulated DEGs in KT1 and in Tri (Table 2). In K14, there were more down-regulated (829) DEGs than up-regulated (789) DEGs only at 6 h (Table 2), and the ratio of down-regulated DEGs constantly decreased from 51.24% (6 h) to 41.22% (12 h) and 39.53% (24 h). As shown in the Venn diagrams, most up-regulated DEGs appeared at 24 h in each line (484, 1,021, and 478, respectively) (Fig 1B–1D, left). Most down-regulated DEGs appeared at 6 h in K14, 12 h in KT1, and 24 h in Tri (Fig 1B–1D, right). At 6 h, KT1 and Tri shared a total of 162 up-regulated and 68 down-regulated genes that were not found in K14 (Fig 1E). At 12 h, KT1 and Tri shared 134 up-regulated and 290 down-regulated genes that were not detected in K14 (Fig 1F). While at 24 h, there were 275 up-regulated and 487 down-regulated genes shared by KT1 and Tri, which were absent in K14 (Fig 1G). These differentially expressed genes were functionally annotated, showing that many genes were related to ABA signaling and calcium ion signaling (S1 Appendix). For example, ABA signaling related genes include “response to abscisic acid” (c92088_graph_c0), “abscisic acid-activated signaling pathway”(c50841_graph_c0), “abscisic acid 8'-hydroxylase” (c80653_graph_c0), “abscisic acid receptor” (c64345_graph_c0), “abscisic acid-activated signaling pathway “(c91458_graph_c0), and “negative regulation of abscisic acid-activated signaling pathway” (c87925_graph_c0), etc (S1 Appendix). Calcium ion signaling related genes include “calcium channel protein” (c85605_graph_c0), “calcium ion binding” (c85877_graph_c0), “calcium-transporting ATPase” (c95423_graph_c0), “calcium uniporter protein” (c80943_graph_c0), and “response to calcium ion” (c68908_graph_c0), etc (S1 Appendix).

**Table 2:** Statistics of differentially expressed genes (DEGs).

	K14			KT1			Tri		
	6 h	12 h	24 h	6 h	12 h	24 h	6 h	12 h	24 h
Up	789(48.76%)	947(58.78%)	1,233(60.47%)	827(47.97%)	1,318(30.92%)	1,946(43.57%)	658(41.91%)	629(43.11%)	815(39.87%)
Down	829(51.24%)	664(41.22%)	806(39.53%)	897(52.03%)	2,945(69.08%)	2,520(56.43%)	912(58.09%)	830(56.89%)	1,229(60.13%)
Total	1,618	1,611	2,039	1,724	4,263	4,466	1,570	1,459	2,044

### Drought-related modules obtained by WGCNA

**Fig 2:**
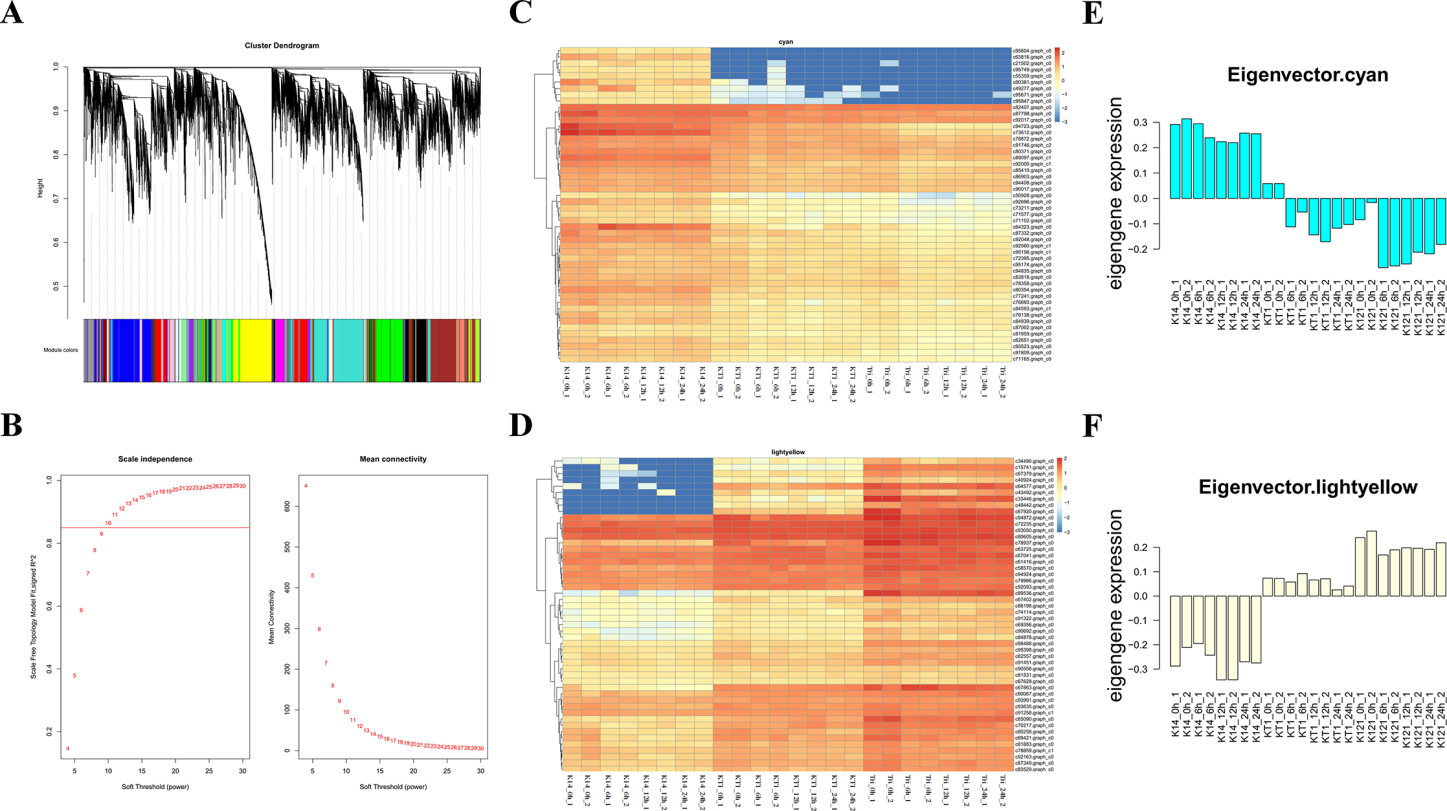
WGCNA module building, Hubgenes cluster analysis, and EigenGene expression statistics. (A, B) Hierarchical clustering of co-expression data. (C) Heatmap cluster of Hubgenes from cyan module. (D) Heatmap cluster of Hubgenes from light yellow module. (E) Eigengenes expression pattern in cyan module. (F) Eigengenes expression pattern in light yellow module.

**Fig 3:**
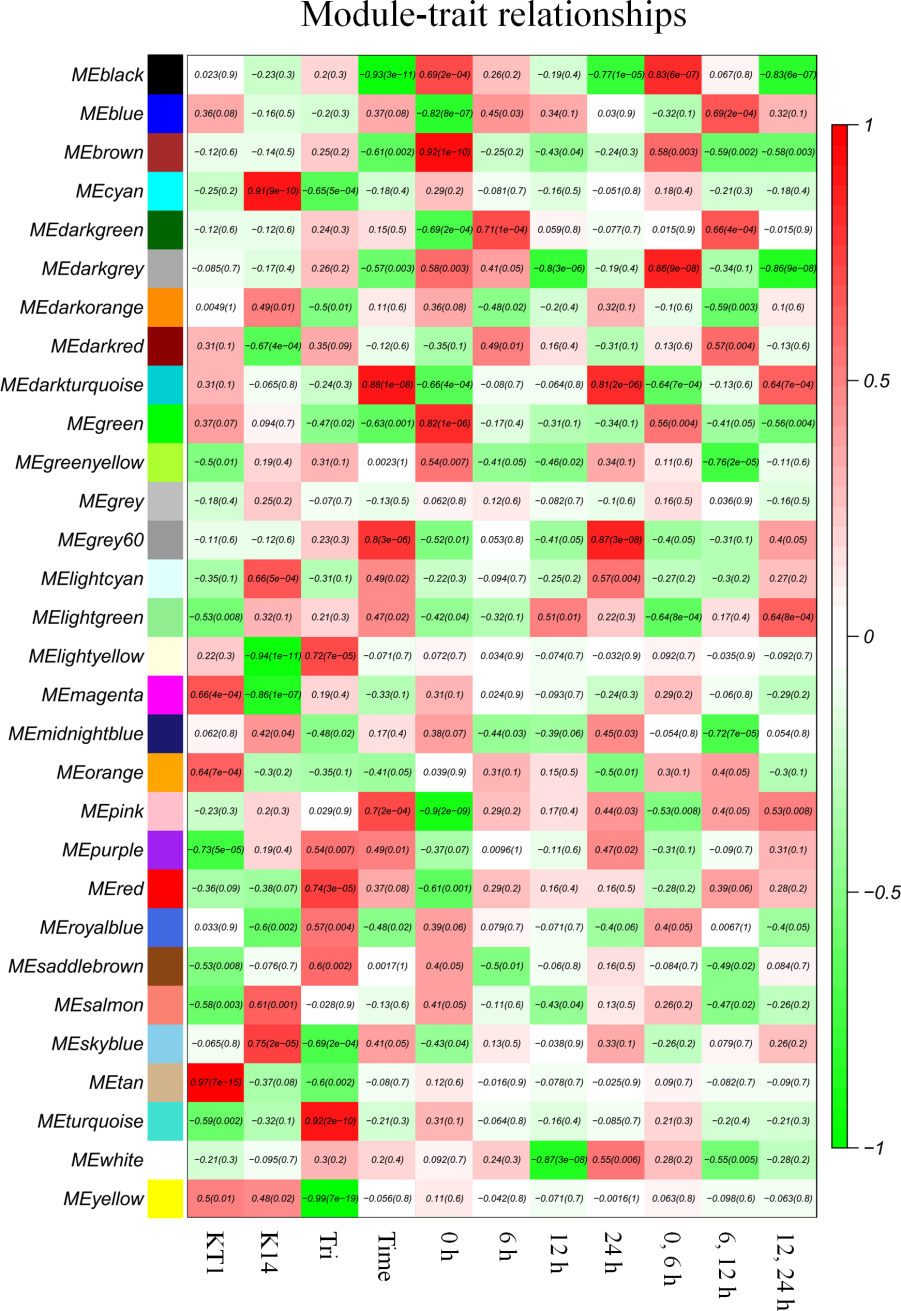
Module correlation coefficients from weighted gene co-expression network analysis. Variables shown on x-axis are genotype (KT1, K14, Tri), time, duration of drought stress treatment (0, 6, 12, 24 h), and difference between two treatment times (0, 6 h; 6, 12 h; 12, 24 h).

The 22,412 unigenes filtered from the above FPKM screening were further analyzed by WGCNA. When constructing the modules, the β parameter was adjusted to 10, yielding a total of 30 modules (Figs 2 and 3, Table 3). Since Tri and KT1 are more drought tolerant than K14, we first screened the modules from Tri with correlation coefficients higher than 0.6. Seven modules were obtained after filtering (Table 4). We further screened the seven modules according to the criterion that the correlation coefficient of a module in KT1 had the same positive or negative property as the same module in Tri, but opposite to that of the same module in K14. In addition, the absolute value of correlation coefficients should not be small. On the basis of these criteria, we chose the cyan and light yellow modules for candidate gene selection (Table 4). The Hubgenes heatmap clustering (Fig 2C and 2D) and the Eigengene expression patterns (Fig 2E and 2F) showed that the expression levels of genes in the cyan module were relatively high in K14, but relatively low in KT1 and Tri. Thus, these drought-associated candidate genes screened from the cyan module might be involved in the negative regulation of drought tolerance. In the light yellow module, gene expression was relatively low in K14 and relatively high in KT1 and Tri. Therefore, the drought-associated candidate genes screened from the light yellow module may be involved in the positive regulation of drought tolerance.

**Table 3:** Number of genes enriched in each module.

Module	Gene numbers	Module	Gene numbers
Black	1,271	Light yellow	309
Blue	2,610	Magenta	504
Brown	2,451	Midnight blue	387
Cyan	420	Orange	111
Dark green	154	Pink	741
Dark grey	140	Purple	489
Dark orange	101	Red	1,499
Dark red	192	Royal blue	229
Dark turquoise	152	Saddle brown	84
Green	2,262	Salmon	434
Green yellow	457	Sky blue	89
Grey	143	Tan	450
Grey60	318	Turquoise	3,267
Light cyan	321	White	89
Light green	312	Yellow	2,305

**Table 4:** Modules with a high correlation coefficient with Tri.

	K14	KT1	Tri
Cyan	0.91 (9e-10)	-0.25 (0.2)	-0.65 (5e-04)
Light yellow	-0.94 (1e-11)	0.22 (0.3)	0.72 (7e-05)
Red	-0.38 (0.07)	-0.36 (0.09)	0.74 (3e-05)
Saddle brown	-0.076 (0.7)	-0.53 (0.008)	0.6 (0.002)
Sky blue	0.75 (2e-05)	-0.065 (0.8)	-0.69 (2e-04)
Turquoise	-0.32 (0.1)	-0.59(0.002)	0.92 (2e-10)
Yellow	0.48 (0.02)	0.5 (0.01)	-0.99 (7e-19)

Note: *P* values are shown in parentheses.

The candidate genes enriched in each module were then ranked according to the level of gene-gene connectivity. The top 20 genes in the cyan and light yellow modules are listed in Tables 5 and 6, respectively. The top 20 genes enriched in the cyan module included those encoding ABA signaling-related (c73612.graph_c0), flowering-related (c71165.graph_c0), disease-related (c60381.graph_c0), and light signal-responsive proteins (c94723.graph_c0, c95174.graph_c0), etc. (Table 5). Among them, c73612.graph_c0 encodes an ABI-like protein involved in reverse regulation of ABA signaling [29–33], and c71165.graph_c0 encodes EMBRYONIC FLOWER 2 (EMF2), which is homologous to the polycomb proteins PRC1 and PRC2. The PRC1 and PRC2 proteins are essential for long-term epigenetic chromatin silencing and for stem cell differentiation and early embryonic development [34, 35].

**Table 5:** Top 20 genes enriched in cyan module.

Unigene ID	Predicted function	Rank	Connection
c92000.graph_c1	Glycogen phosphorylase 1-like	1	29.87649
c73612.graph_c0	Protein ABIL1	2	28.92155
c92048.graph_c0	Putative ribonuclease H protein At1g65750 GN = At1g65750	3	28.88248
c80354.graph_c0	Uncharacterized protein LOC102614759	4	28.7481
c89097.graph_c1	Molecular Function: DNA binding	5	28.21814
c71165.graph_c0	Polycomb group protein EMBRYONIC FLOWER 2 isoform X2	6	28.17468
c92696.graph_c0	Gag-protease-integrase-RT-RNaseH polyprotein	7	27.42639
c71577.graph_c0	Uncharacterized protein LOC104224935	8	26.02592
c60381.graph_c0	Putative late blight resistance protein homolog R1B-16	9	25.62843
c92560.graph_c1	Integrase core domain containing protein	10	25.31057
c93523.graph_c0	Uncharacterized protein LOC104243519 isoform X4	11	25.2015
c78358.graph_c0	Putative ribonuclease H protein At1g65750 GN = At1g65750	12	25.04837
c73211.graph_c0	Probable beta-1,3-galactosyltransferase 2	13	25.01559
c94723.graph_c0	Protein FAR1-RELATED SEQUENCE 5-like	14	24.95755
c95174.graph_c0	Protein FAR1-RELATED SEQUENCE 6-like	15	24.74544
c86903.graph_c0	Phosphatidate cytidylyltransferase, mitochondrial isoform X1	16	24.56781
c83818.graph_c0	Zinc finger BED domain-containing protein RICESLEEPER 1-like	17	24.19857
c92017.graph_c0	Serrate RNA effector molecule-like isoform X2	18	24.14518
c77241.graph_c0	Equilibrative nucleotide transporter 3	19	23.37428
c87332.graph_c0	Replication, recombination and repair	20	23.05712

**Table 6:** Top 20 genes enriched in light yellow module.

Unigene ID	Predicted function	Rank	Connection
c33446.graph_c0	Ribonuclease-like storage protein (Precursor)	1	30.16488
c67920.graph_c0	Putative transposase	2	27.96056
c78937.graph_c0	Peptidyl-prolyl cis-trans isomerase FKBP15-1	3	27.94231
c85090.graph_c0	Male gamete fusion factor	4	27.71013
c91322.graph_c0	Putative calcium-transporting ATPase 13, plasma membrane-type GN = ACA13	5	27.56307
c87349.graph_c0	Biological Process: regulation of nucleobase-containing compound metabolic process	6	27.26889
c49442.graph_c0	Zinc finger CCCH domain-containing protein 20-like	7	26.93657
c67402.graph_c0	Uncharacterized protein LOC104236329, partial	8	26.78864
c88488.graph_c0	Pentatricopeptide repeat-containing protein At3g26782, mitochondrial	9	26.59128
c91258.graph_c1	Protein ROOT PRIMORDIUM DEFECTIVE 1	10	26.46877
c83529.graph_c0	Uncharacterized protein LOC104114770 isoform X1	11	26.0098
c40924.graph_c0	TMV resistance protein N-like	12	25.973
c80605.graph_c0	DDE superfamily endonuclease	13	25.26208
c89536.graph_c0	Putative transposase	14	24.78359
c69356.graph_c0	Hypothetical protein MIMGU_mgv1a008090mg	15	24.66299
c67379.graph_c0	Unnamed protein product	16	24.66229
c94924.graph_c0	ATP-dependent RNA helicase DHX36 isoform X2	17	24.62971
c90067.graph_c0	Cysteine synthase	18	24.35074
c58570.graph_c0	Hypothetical protein CISIN_1g0242011mg, partial	19	24.31653
c70217.graph_c0	Pyridoxamine 5'-phosphate oxidase	20	23.29847

The screening also identified genes encoding DNA binding (c89097.graph_c1) or DNA replication (c92048.graph_c0, c92696.graph_c0, c78358.graph_c0, c87332.graph_c0) proteins. The transcript levels of these genes decreased under drought stress (S1 Fig), indicating that DNA replication and cell proliferation decelerated in K14, KT1, and Tri during the response to drought stress (S1 Fig). The top 20 genes enriched in the light yellow module included those encoding a ribonuclease-like storage protein (c33446.graph_c0), a calcium-transporting ATPase (Ca^2+^-ATPase, c91322.graph_c0), a zinc finger CCCH domain-containing protein (c49442.graph_c0), a pentatricopeptide repeat-containing (PPR) protein (c88488.graph_c0), and ROOT PRIMORDIUM DEFECTIVE 1 (c91258.graph_c0), etc (Table 6, S2 Fig).

### Cloning of candidate genes and evolutionary analysis

**Fig 4:**
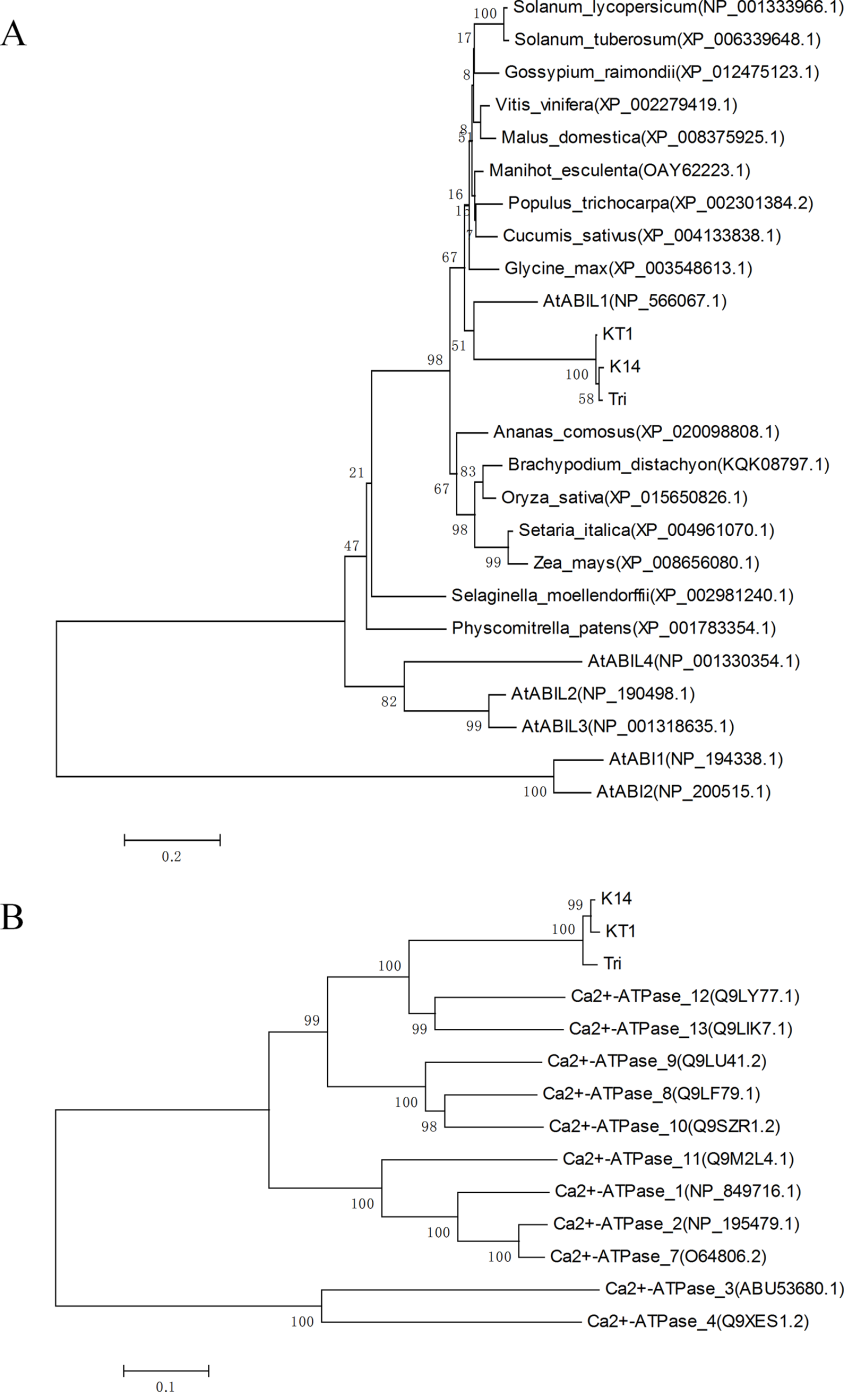
Phylogenetic analysis of ABI-like and Ca^2+^ ATPase genes. (A) Evolutionary analysis of ABI-like genes in K14, KT1, and Tri with their homologs from different plant species and with ABI proteins from Arabidopsis. (B) Evolutionary analysis of Ca^2+^-ATPase genes in K14, KT1, and Tri with Arabidopsis homologs. Length of branch lines indicates the extent of divergence.

Two candidate genes identified in the WGCNA, ABI-like protein (c73612.graph_c0) and Ca^2+^-ATPase (c91322.graph_c0), were further analyzed. The open reading frame (ORF) of these two genes were amplified and their sequences could be found in S2 Appendix. Both the ABI-like protein and Ca^2+^-ATPase belong to gene families and previous studies have demonstrated they were major players in drought tolerance [29–33, 36, 37]. The high ranking of these two genes in the gene-gene connectivity analysis also indicated that they might play important roles in drought tolerance. Their evolutionary relationships with homologous genes from different plant species are shown in Fig 4, which showed that ABI-like genes of K14, KT1 and Tri had higher homology with AtABIL1. Moreover, they are more closely related to homologs from dicots rather than to homologs from monocots (Fig 4A). In addition, Ca^2+^-ATPase genes of K14, KT1 and Tri were more homologous to Ca^2+^-ATPase 12 and 13 from Arabidopsis (Fig 4B).

## Discussion

### High-quality transcriptome data lay a foundation for further research

The lack of a reference genome for sweetpotato seriously restricts research on the molecular mechanisms of this species and its close relatives. Although previous studies have reported transcriptome sequencing of sweetpotato, the numbers of transcripts and unigenes were relatively small due to the small size of sequencing samples. In addition, the splicing length was relatively short [38, 39]. In this study, RNA from 24 whole plants was sequenced, with high-quality sequence splicing and many more genes obtained than in previous studies (Table 1). Finally, 322,803 transcripts and 105,959 unigenes were assembled (N50 of 1,965 and 1,403, respectively). Of the transcripts and unigenes, 148,263 (83,496 + 64,767) transcripts and 23,083 (13,328 + 9,755) unigenes were longer than 1 kb (Table 1). These sequencing results lay the foundation for further research on the molecular mechanisms of sweetpotato and its close relatives.

### Cyan and light yellow modules are most related to drought tolerance

Several studies have demonstrated that WGCNA is a powerful tool to screen transcriptome sequencing and chip detection data for key candidate genes [18, 40, 41]. Firstly, WGCNA provides various modules, which are groups of genes possibly related to phenotypic differences. Modules can then be further screened to find candidate genes. Reasonable selection combined with phenotypic trait data can identify the most closely related modules. Our previous work showed that Tri and KT1 (which inherited some genetic material from Tri) are strongly drought tolerant, whereas K14 is not [9, 10]. Based on these phenotypic characteristics, we first chose the modules with a high correlation coefficient in Tri. There were seven modules with absolute values of ≥ 0.6. Secondly, we compared the correlation coefficients of these modules in K14 and KT1 with those in Tri. We expected that the correlation coefficients related to KT1 and Tri would be both positive or negative, and the correlation coefficient related to K14 should be the opposite. In addition, the absolute value of the correlation coefficients should not be small. The cyan and light yellow modules met these requirements (Table 4).

### ABI-like protein is an important negative regulator responsible for drought tolerance in KT1 and Tri

The Hubgenes heatmap clustering and the Eigengenes expression patterns showed that, in the cyan module, the overall gene expression level was high in K14 but low in KT1 and Tri (Fig 2C and 2E). This suggests that negative regulators associated with drought tolerance could be screened from this module. Genes encoding proteins related to ABA signaling (c73612.graph_c0), flowering (c71165.graph_c0), disease (c60381.graph_c0), and light signal responsiveness (c94723.graph_c0, c95174.graph_c0) were enriched in the top 20 candidate genes in this module (Table 5).

Several studies have shown that ABA signaling is related to drought tolerance [42–45]. In plants, ABA is an endogenous hormone that plays important roles in growth and development, and in the responses to abiotic stresses such as high salinity, drought, and low temperature. Under stress conditions, plants increase ABA content by regulating its biosynthesis and transport. The changes in ABA content induce stomatal closure, promote the accumulation of certain substances, and regulate the expression of stress-related genes [29–33]. The ABA content in plants is also associated with ABA insensitive (ABI) factors. In Arabidopsis, ABI1-5 and other PP2C proteins are accessory proteins that diminish ABA signaling by inhibiting downstream protein kinases. ABA binds to its receptor protein PYR/PYL/RCAR to inhibit PP2Cs protein activity, thereby activating ABA signaling [46–52]. Evolutionally, ABI-like proteins diverged from ABIs. However, Arabidopsis ABI1-like 1 (ABIL1) appears to mediate similar interactions as ABI1 that assembles DIS3/SCAR2 into a SCAR/WAVE complex [53–54]. Components of SCAR/WAVE complex are revealed to mediate epidermal cell morphogenesis [55], stomatal response [56] and water loss [57]. In the cyan module, c73612.graph_c0, encoding an ABI-like protein, was highly ranked in the gene-gene connection analysis (Table 5). The c73612.graph_c0 transcript levels were low in Tri and KT1 but high in K14, consistent with the differences in their drought-tolerance phenotypes (S1 Fig). We speculate that the low expression level may contribute to drought tolerance in Tri and KT1 by releasing the inhibition of ABA signaling. Therefore, we suggest that the ABI-like protein acts as an important negative regulator in the drought tolerance of Tri and KT1.

### Ca^2+^-ATPase is an important positive regulator of drought tolerance in KT1 and Tri

The Hubgenes heatmap clustering and the Eigengenes expression patterns showed that in the light yellow module, the overall gene expression level was low in K14 but high in KT1 and Tri (Fig 2D and 2F). This result indicated that positive regulators of drought tolerance could be screened from this module. Among the top 20 genes enriched in the light yellow module, CCCH-type zinc finger proteins are associated with mRNA destabilization [58]. PPR is a 35-amino acid motif and some proteins containing PPR locate in organelles such as mitochondria and express in guard cells and seeds [59, 60]. Arabidopsis CRR4 containing PPR participates in RNA editing [61]. ROOT PRIMORDIUM DEFECTIVE 1 (RPD1) is a plant-specific gene that is required for the proliferation of active cells. It has been shown to play a role in generating adventitious roots in response to exogenous auxins in Arabidopsis hypocotyls. Arabidopsis *rpd1* mutants are susceptible to temperature variations, and high temperature (28°C) was shown to hinder root primordium development [62].

While Ca^2+^-ATPase is involved in transporting Ca^2+^, which is an important second messenger in plants and an important component of plants’ responses to various stresses. The Ca^2+^ signaling pathway plays an important role in responses to biotic and abiotic stresses including pathogen and pest attacks, salt, drought, and low or high temperatures [63]. An increase in the cytoplasmic Ca^2+^ concentration is an important step in early ABA signaling [48]. Silencing of the Ca^2+^-binding protein SCaBP5 and its interacting protein PKS3 in Arabidopsis rendered seed germination, seedling growth, stomatal closure, and gene expression sensitive to ABA [64]. Ca^2+^-ATPases participate in Ca^2+^ signal regulation. For example, the plasma membrane Ca^2+^-ATPase (PMCA) is a cytoplasmic membrane transporter that removes Ca^2+^ from cells [65]. Therefore, proteins in the Ca^2+^-ATPase family play an important role in plants’ environmental stress responses. Overexpression of *GsACA1* in *Glycine soja* significantly improved its tolerance to carbonic acid and alkaline salt stresses [36, 37].

In the light yellow module, the Ca^2+^-ATPase gene c91322.graph_c0 ranked highly (fifth) in the gene-gene connection analysis (Table 6). Its transcript abundance was highest in Tri, followed by KT1, but was very low in K14. In addition, the changes in the transcript abundance of c91322.graph_c0 during drought stress were very similar in Tri and KT1 (significant decrease at 6h, followed by a slow increase; S2 Fig). Based on the above analyses, we speculate that Ca^2+^-ATPase is an important positive regulator of drought tolerance in Tri and KT1.

## Conclusions

The drought-stress responses of the sweetpotato somatic hybrid KT1 and its parents K14 and Tri were compared by transcriptome sequencing analyses. Gene transcript levels were analyzed and DEGs were screened. A total of 22,412 unigenes were analyzed by WGCNA to select negatively or positively regulatory modules, and key candidate genes were identified. Further research should focus on functional analyses of these candidate genes during the drought response.

## Supporting information

S1 Fig Transcript abundance values (log_2_ (FPKM+1)) of the top 20 genes enriched in cyan module according to connection degree. Values shown are the averages of two biological replicates.
(TIF)

S2 Fig Transcript abundance values (log_2_ (FPKM+1)) of the top 20 genes enriched in light yellow module according to connection degree. Values shown are the averages of two biological replicates.
(TIF)

S1 Appendix Functional annotations of differentially expressed genes. (XLSX)

S2 Appendix ORF sequences of ABI-like protein gene and Ca^2+^-ATPase gene in K14, KT1, and Tri. (TXT)

S3 Appendix Quality assessment and functional annotation of the transcriptome data. (DOCX)

S1 Table Transcriptome sequencing statistics. (DOCX)

S2 Table Statistics of annotated unigenes. (DOCX)

S3 Table PCR primers used to amplify candidate genes. (DOCX)
