# Supplementary figures and images for: Involvement of an ABI-like protein and a Ca2+-ATPase in drought tolerance as revealed by transcript profiling of a sweetpotato somatic hybrid and its parents Ipomoea batatas (L.) Lam. and I. triloba L
Source: PLoS One. 2018 Feb 21;13(2):e0193193. doi: 10.1371/journal.pone.0193193 (PMC5821372; doi:10.1371/journal.pone.0193193)

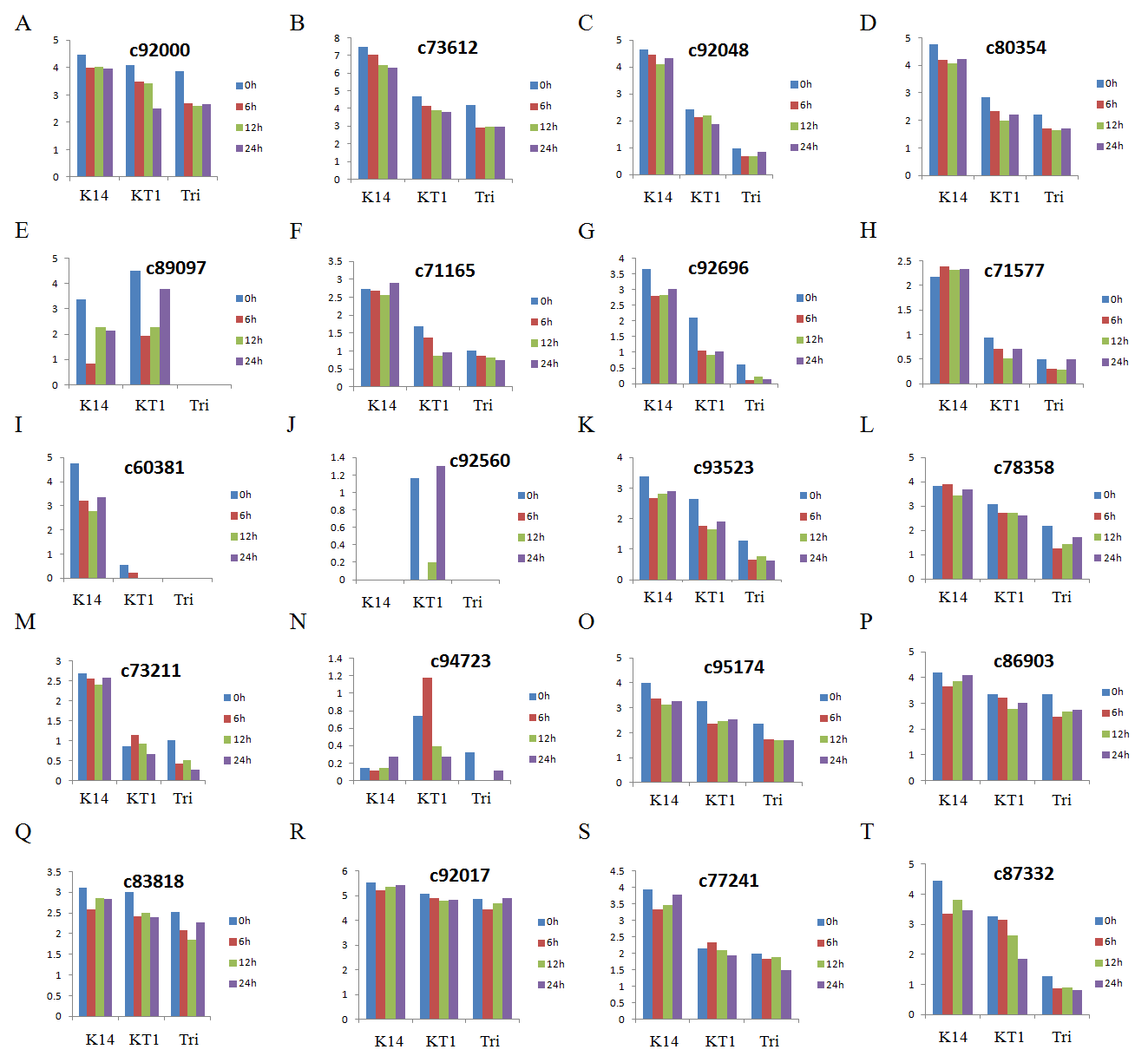

Supplement: S1 Fig — Values shown are the averages of two biological replicates. (TIF) [file pone.0193193.s001.tif]

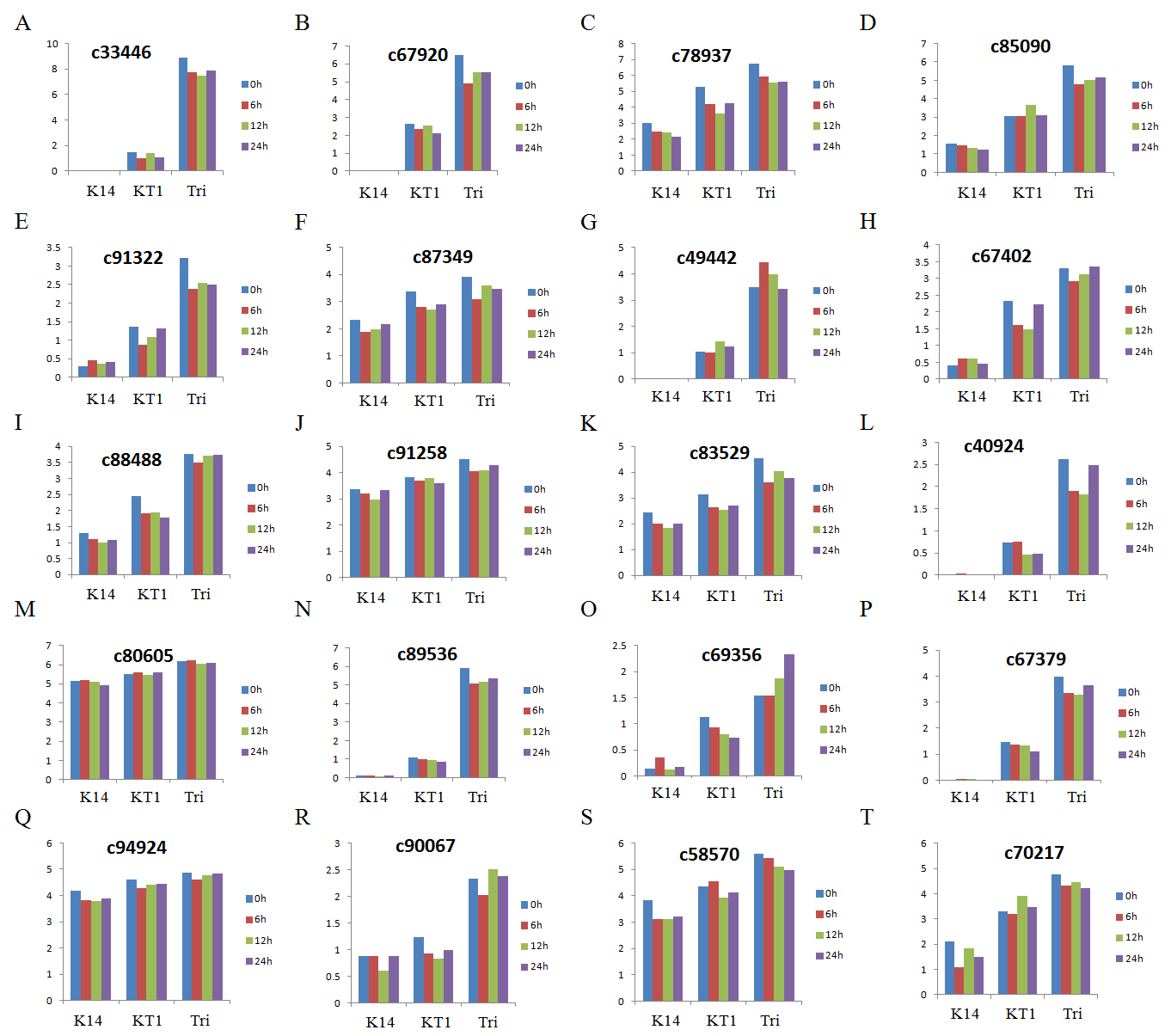

Supplement: S2 Fig — Values shown are the averages of two biological replicates. (TIF) [file pone.0193193.s002.tif]
