# Supplementary material for: Involvement of an ABI-like protein and a Ca2+-ATPase in drought tolerance as revealed by transcript profiling of a sweetpotato somatic hybrid and its parents Ipomoea batatas (L.) Lam. and I. triloba L
Source: PLoS One. 2018 Feb 21;13(2):e0193193. doi: 10.1371/journal.pone.0193193 (PMC5821372; doi:10.1371/journal.pone.0193193)
Supplement: S3 Appendix — (DOCX) [file pone.0193193.s005.docx]

Table 1. Genetic mapping of clean transcriptomic data with *I. trifida* (assembly ITR_r1.0) after quality control

| Sample ID | Sample | Total reads | Unique mapped | Multiple mapped | Total mapped |
| --- | --- | --- | --- | --- | --- |
| A1 | K14-1-0h | 26,082,540 | 46.54% | 8.02% | 54.56% |
| A2 | K14-2-0h | 25,666,945 | 45.41% | 8.74% | 54.15% |
| A3 | KT1-1-0h | 27,405,295 | 45.82% | 7.48% | 53.30% |
| A4 | KT1-2-0h | 34,012,042 | 45.56% | 7.10% | 52.66% |
| A5 | K121-1-0h | 26,390,155 | 48.86% | 6.42% | 55.28% |
| A6 | K121-2-0h | 31,862,015 | 49.64% | 6.33% | 55.97% |
| A7 | K14-1-6h | 36,599,310 | 51.48% | 7.03% | 58.51% |
| A8 | K14-2-6h | 29,941,093 | 51.25% | 7.03% | 58.28% |
| A9 | KT1-1-6h | 28,172,802 | 51.38% | 6.84% | 58.22% |
| A10 | KT1-2-6h | 29,676,347 | 52.03% | 6.56% | 58.59% |
| A11 | K121-1-6h | 24,146,171 | 50.27% | 6.68% | 56.95% |
| A12 | K121-2-6h | 24,452,354 | 49.53% | 6.25% | 55.78% |
| A13 | K14-1-12h | 24,763,050 | 47.03% | 6.83% | 53.86% |
| A14 | K14-2-12h | 26,816,396 | 47.46% | 7.16% | 54.62% |
| A15 | KT1-1-12h | 28,089,236 | 51.98% | 6.05% | 58.03% |
| A16 | KT1-2-12h | 28,417,473 | 53.09% | 5.76% | 58.85% |
| A17 | K121-1-12h | 28,182,547 | 48.73% | 5.39% | 54.12% |
| A18 | K121-2-12h | 28,025,529 | 49.02% | 5.80% | 54.82% |
| A19 | K14-1-24h | 28,871,187 | 47.26% | 7.86% | 55.12% |
| A20 | K14-2-24h | 27,260,019 | 47.45% | 7.90% | 55.35% |
| A21 | KT1-1-24h | 26,051,909 | 46.68% | 6.62% | 53.30% |
| A22 | KT1-2-24h | 25,702,955 | 47.49% | 6.70% | 54.19% |
| A23 | K121-1-24h | 33,011,170 | 50.78% | 6.30% | 57.08% |
| A24 | K121-2-24h | 27,623,890 | 50.46% | 6.43% | 56.89% |
| Mean |  | 28,217,601.25 | 48.97% | 6.80% | 55.77% |

Table 2. BLAST searches of genes from the cyan module in Araport11 database (Release_201606)

| Unigene ID | Arabidopsis Loci | Function Description | E value |
| --- | --- | --- | --- |
| c92000.graph_c1 | AT3G46970.1 | Alpha-glucan phosphorylase 2 | 0.0 |
| c73612.graph_c0 | AT2G46225.1 | ABI-1-like 1 | 2e-062 |
| c92048.graph_c0 | AT4G29090.1 | Ribonuclease H-like superfamily protein | 2e-081 |
| c80354.graph_c0 | AT5G09580.1 | Heat shock protein | 9e-072 |
| c89097.graph_c1 | AT1G53860.2 | Remorin family protein | 3e-005 |
| c71165.graph_c0 | AT5G51230.1 | VEFS-Box of polycomb protein | 2e-104 |
| c92696.graph_c0 | AT4G23160.3 | Cysteine-rich RECEPTOR-like kinase | 1e-112 |
| c71577.graph_c0 | AT3G54880.1 | Zinc finger protein | 4e-019 |
| c60381.graph_c0 | AT1G50180.1 | NB-ARC domain-containing disease resistance protein | 5e-016 |
| c92560.graph_c1 | AT1G11790.1 | Arogenate dehydratase 1 | 6e-045 |
| c93523.graph_c0 | AT1G58210.1 | EMBRYO DEFECTIVE protein | 4e-007 |
| c78358.graph_c0 | AT4G29090.1 | Ribonuclease H-like superfamily protein | 3e-074 |
| c73211.graph_c0 | AT1G05170.2 | Galactosyltransferase family protein | 2e-006 |
| c94723.graph_c0 | AT4G38180.1 | FAR1-related sequence 5 | 2e-070 |
| c95174.graph_c0 | AT1G52520.1 | FAR1-related sequence 6 | 0.0 |
| c86903.graph_c0 | AT3G47630.6 | Translocator assembly/maintenance protein | 1e-053 |
| c83818.graph_c0 | AT3G42170.2 | BED zinc finger and hAT dimerization domain-containing  protein DAYSLEEPER | 4e-053 |
| c92017.graph_c0 | AT2G27100.1 | C2H2 zinc-finger protein SERRATE (SE) | 0.0 |
| c77241.graph_c0 | AT4G05110.3 | Equilibrative nucleoside transporter 6 | 0.0 |
| c87332.graph_c0 | AT1G42190.1 | GAG/POL/ENV polyprotein | 0.17 |

Table 3. BLAST search of genes from the light-yellow module in Araport11 database (Release_201606)

| Unigene ID | Arabidopsis Loci | Function Description | E value |
| --- | --- | --- | --- |
| c33446.graph_c0 | AT1G14220.1 | Ribonuclease T2 family protein | 2e-022 |
| c67920.graph_c0 | AT2G05642.1 | Nucleic acid-binding%2C OB-fold-like protein | 6e-006 |
| c78937.graph_c0 | AT3G25220.1 | FK506-binding protein 15 kD-1 | 1e-072 |
| c85090.graph_c0 | AT4G11720.1 | Hapless 2 | 9e-139 |
| c91322.graph_c0 | AT3G22910.1 | ATPase E1-E2 type family protein / haloacid dehalogenase-like  hydrolase family protein | 0.0 |
| c87349.graph_c0 | AT5G48310.2 | Portal protein | 8e-051 |
| c49442.graph_c0 | AT2G19810.1 | CCCH-type zinc finger family protein | 8e-032 |
| c67402.graph_c0 | AT1G06740.1 | MuDR family transposase | 2e-013 |
| c88488.graph_c0 | AT3G26782.1 | Tetratricopeptide repeat (TPR)-like superfamily  protein | 0.0 |
| c91258.graph_c1 | AT3G63090.1 | Ubiquitin carboxyl-terminal hydrolase family protein | 2e-151 |
| c83529.graph_c0 | AT3G17030.1 | Nucleic acid-binding proteins superfamily | 5e-120 |
| c40924.graph_c0 | AT5G36930.2 | Disease resistance protein (TIR-NBS-LRR class) | 1e-024 |
| c80605.graph_c0 | AT3G50360.1 | Centrin2 | 5e-055 |
| c89536.graph_c0 | AT2G05642.1 | Nucleic acid-binding%2C OB-fold-like protein | 0.98 |
| c69356.graph_c0 | AT5G49610.1 | F-box family protein | 9e-009 |
| c67379.graph_c0 | AT5G53440.2 | LOW protein: zinc finger CCCH domain protein | 2e-055 |
| c94924.graph_c0 | AT3G26560.1 | ATP-dependent RNA helicase | 7e-040 |
| c90067.graph_c0 | AT5G28020.6 | Cysteine synthase D2 | 3e-147 |
| c58570.graph_c0 | AT3G11630.1 | Thioredoxin superfamily protein | 1e-040 |
| c70217.graph_c0 | AT2G04690.1 | Pyridoxamine 5'-phosphate oxidase family protein | 3e-076 |


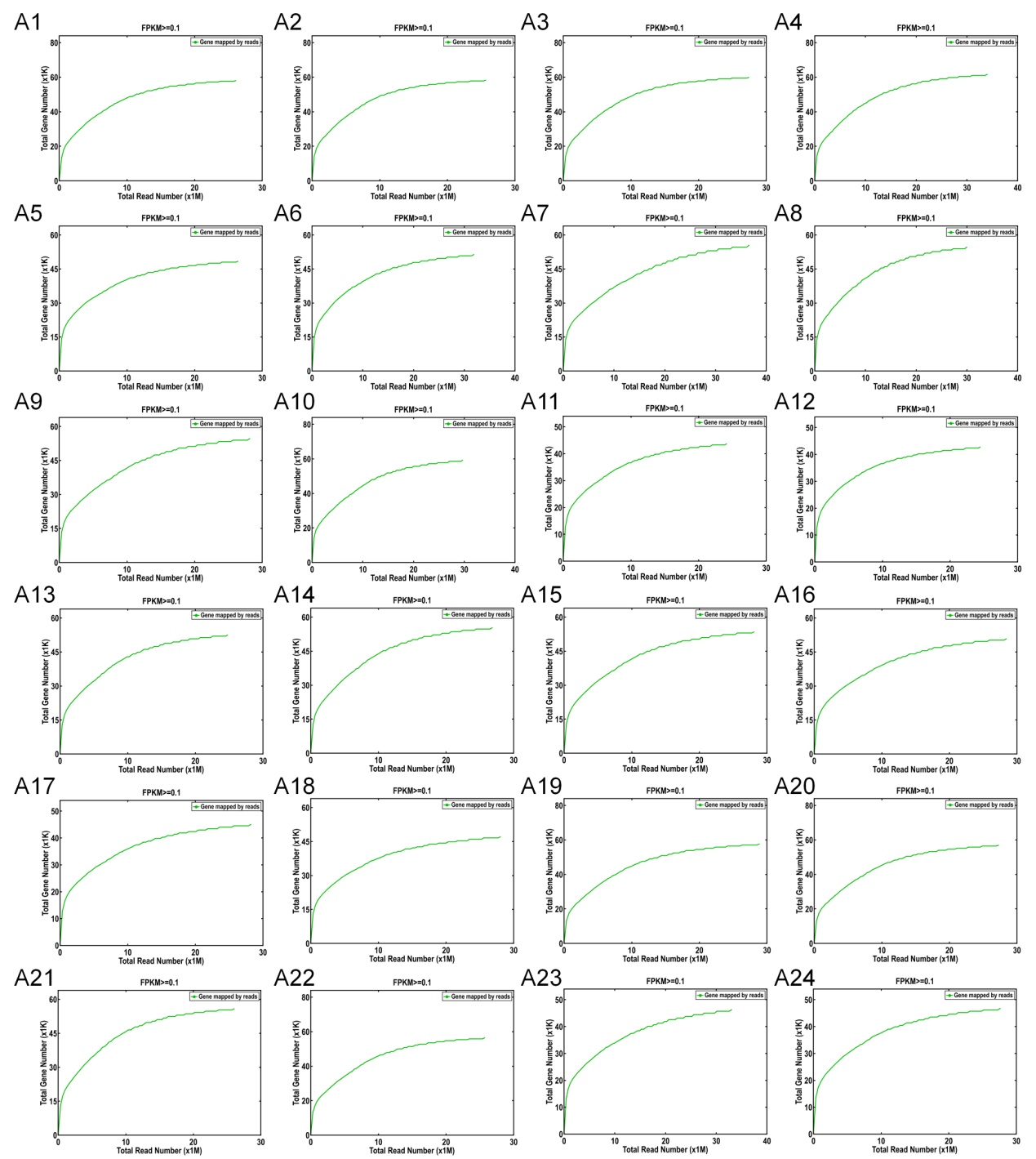


Figure 1. Saturation simulation plot of the transcriptome data
