# Supplementary material for: Involvement of an ABI-like protein and a Ca2+-ATPase in drought tolerance as revealed by transcript profiling of a sweetpotato somatic hybrid and its parents Ipomoea batatas (L.) Lam. and I. triloba L
Source: PLoS One. 2018 Feb 21;13(2):e0193193. doi: 10.1371/journal.pone.0193193 (PMC5821372; doi:10.1371/journal.pone.0193193)
Supplement: S1 Table — (DOCX) [file pone.0193193.s006.docx]

Supplemental Table 1. Transcriptome sequencing statistics

| **Samples** | **Read number** | **Base number** | **GC content** | **%≥Q30** |
| --- | --- | --- | --- | --- |
| A1 | 26,082,540 | 6,570,741,922 | 46.80% | 89.80% |
| A2 | 25,666,945 | 6,466,199,814 | 46.97% | 90.31% |
| A3 | 27,405,295 | 6,904,444,242 | 46.74% | 90.43% |
| A4 | 34,012,042 | 8,567,634,489 | 46.82% | 90.08% |
| A5 | 26,390,155 | 6,648,624,643 | 45.99% | 89.50% |
| A6 | 31,862,015 | 8,026,421,740 | 45.73% | 90.00% |
| A7 | 36,599,310 | 9,218,645,089 | 45.85% | 89.93% |
| A8 | 29,941,093 | 7,542,214,523 | 46.11% | 89.98% |
| A9 | 28,172,802 | 7,096,381,985 | 46.08% | 89.96% |
| A10 | 29,676,347 | 7,475,480,867 | 45.82% | 90.15% |
| A11 | 24,146,171 | 6,082,785,070 | 46.50% | 92.08% |
| A12 | 24,452,354 | 6,160,704,079 | 46.49% | 91.83% |
| A13 | 24,763,050 | 6,238,717,777 | 47.47% | 92.37% |
| A14 | 26,816,396 | 6,755,603,291 | 47.17% | 93.00% |
| A15 | 28,089,236 | 7,076,076,405 | 45.65% | 92.02% |
| A16 | 28,417,473 | 7,158,407,755 | 45.66% | 91.81% |
| A17 | 28,182,547 | 7,100,187,188 | 46.02% | 91.93% |
| A18 | 28,025,529 | 7,060,559,685 | 45.90% | 92.06% |
| A19 | 28,871,187 | 7,274,166,852 | 47.11% | 89.89% |
| A20 | 27,260,019 | 6,868,006,177 | 47.13% | 89.99% |
| A21 | 26,051,909 | 6,564,039,804 | 47.27% | 92.08% |
| A22 | 25,702,955 | 6,475,650,739 | 47.03% | 92.92% |
| A23 | 33,011,170 | 8,314,427,514 | 46.41% | 88.22% |
| A24 | 27,623,890 | 6,957,996,691 | 46.12% | 91.38% |
| Average | 28,217,601.25 | 7,108,504,930.86 | 46.45% | 90.91% |
