# Supplementary material for: Involvement of an ABI-like protein and a Ca2+-ATPase in drought tolerance as revealed by transcript profiling of a sweetpotato somatic hybrid and its parents Ipomoea batatas (L.) Lam. and I. triloba L
Source: PLoS One. 2018 Feb 21;13(2):e0193193. doi: 10.1371/journal.pone.0193193 (PMC5821372; doi:10.1371/journal.pone.0193193)
Supplement: S2 Table — (DOCX) [file pone.0193193.s007.docx]

Supplemental Table 2. Statistics of annotated unigenes

| **Anno_database** | **Annotated_number** | **300 ≤ Length < 1000** | **Length ≥ 1000** |
| --- | --- | --- | --- |
| COG_Annotation | 9,775 | 2,086 | 6,721 |
| GO_Annotation | 18,728 | 5,943 | 10,232 |
| KEGG_Annotation | 11,591 | 3,521 | 6,808 |
| KOG_Annotation | 20,258 | 6,220 | 11,528 |
| Pfam_Annotation | 24,683 | 6,719 | 15,592 |
| Swissprot_Annotation | 23,582 | 7,244 | 13,802 |
| nr_Annotation | 35,990 | 11,878 | 19,238 |
| All_Annotated | 36,767 | 12,170 | 19,296 |
