# Supplementary material for: Involvement of an ABI-like protein and a Ca2+-ATPase in drought tolerance as revealed by transcript profiling of a sweetpotato somatic hybrid and its parents Ipomoea batatas (L.) Lam. and I. triloba L
Source: PLoS One. 2018 Feb 21;13(2):e0193193. doi: 10.1371/journal.pone.0193193 (PMC5821372; doi:10.1371/journal.pone.0193193)
Supplement: S3 Table — (DOCX) [file pone.0193193.s008.docx]

Supplemental Table 3. PCR primers used to amplify candidate genes

| **Primer** | **Sequence** | **Reference** |
| --- | --- | --- |
| c73612-F1 | 5’-ATGGAGGTGCAGAAAACGAAGC-3’ | c73612.graph_c0 |
| c73612-R1 | 5’-TTATGTCATTGCAGTACCAGTCTTC-3’ | c73612.graph_c0 |
| c91322-F1 | 5’-ATGGCCCGAGCTTCACCA-3’ | c91322.graph_c0 |
| c91322-R1 | 5’-CTAGGCACTGTATTCACGAGAAAGA-3’ | c91322.graph_c0 |
